# Kinin B1 receptor blockade attenuates hepatic fibrosis and portal hypertension in chronic liver diseases in mice

**DOI:** 10.1186/s12967-022-03808-7

**Published:** 2022-12-13

**Authors:** Dileep Reddy Rampa, Huiying Feng, Sivakumar Allur-Subramaniyan, Kwanseob Shim, Anton Pekcec, Dongwon Lee, Henri Doods, Dongmei Wu

**Affiliations:** 1 Department of Bio-Nanotechnology and Bio-Convergence Engineering, Jeonbuk National University, Jeonju, South Korea; 2 Department of Animal Biotechnology & Agricultural Convergence Technology, Jeonbuk National University, Jeonju, South Korea; 3 Research Beyond Borders, Boehringer Ingelheim Pharma GmbH & Co. KG, Biberach, Germany; 4 Department of Research, Mount Sinai Medical Center, Miami Beach, FL USA

**Keywords:** B1R, BI 113823, Hepatic inflammation, Hepatic fibrosis, Portal hypertension, PI3K/AKT signalling pathway

## Abstract

**Background and aims:**

Kinin B1 receptors (B1Rs) are implicated in the pathogenesis of fibrosis. This study examined the anti-fibrotic effects of B1R blockade with BI 113823 in two established mouse models of hepatic fibrosis induced by intraperitoneal carbon tetrachloride (CCl_4_) injection or bile duct ligation (BDL). The mechanisms underlying the protection afforded by B1R inhibition were examined using human peripheral blood cells and LX2 human hepatic stellate cells (HSCs).

**Methods:**

Fibrotic liver diseases were induced in mice by intraperitoneal carbon tetrachloride (CCl_4_) injection for 6 weeks, and by bile duct ligation (BDL) for 3 weeks, respectively. Mice received daily treatment of vehicle or BI 113823 (B1R antagonist) from onset of the experiment until the end of the study.

**Results:**

B1Rs were strongly induced in fibrotic mouse liver. BI 113823 significantly attenuated liver fibrosis and portal hypertension (PH), and improved survival in both CCl_4_ and BDL mice. BI 113823 significantly reduced the expression of fibrotic proteins α-SMA, collagens 1, 3, 4, and profibrotic growth factors PDGF, TGFβ, CTGF, VEGF, proliferating cell nuclear antigen; and reduced hepatic Akt phosphorylation in CCl_4_- and BDL-induced liver fibrosis. BI 113823 also reduced expression of Cytokines IL-1, IL-6; chemokines MCP-1, MCP-3 and infiltration of inflammatory cells; and inhibited human monocyte and neutrophil activation, transmigration, TNF-α & MPO production in vitro. BI 113823 inhibited TGF-β and B1R agonist-stimulated human-HSC activation, contraction, proliferation, migration and fibrosis protein expression, and inhibited activation of PI3K/Akt signalling pathway.

**Conclusions:**

B1Rs merits consideration as a novel therapeutic target for chronic liver fibrosis and PH.

**Supplementary Information:**

The online version contains supplementary material available at 10.1186/s12967-022-03808-7.

## Introduction

Liver fibrosis is a progressive pathological process characterized by excessive deposition of extracellular matrix (ECM) proteins. This is resulting from a dynamic process of wound-healing in response to chronic liver injury triggered by a variety of causes, including alcohol abuse, viral infection, metabolic factors and bile disorders [1, 2]. If the underlying injury persists, fibrosis may progress to its end stage cirrhosis, the major determinant of morbidity and mortality in patients with chronic liver disease (CLD), predisposing to life-threatening complications of portal hypertension (PH) and liver failure [1–3]. Liver fibrosis is usually initiated by hepatocyte injury, resulting in the recruitment of inflammatory cells along with activation of hepatic stellate cells (HSCs) from quiescent cells to proliferating, contractile myofibroblasts, a pivotal event during fibrogenesis, and activation of Kupffer cells [4, 5]. Activated HSCs and myofibroblasts express alpha-1 smooth muscle actin (α-SMA), produce large amounts of collagen, ECM proteins, immunomodulatory cytokines and growth factors, therefore, targeting components of this multi-factorial process may represent an effective approach for future treatment of CLD [1–5].

Kinins are pro-inflammatory peptides that exert a wide range of biological effects via stimulation of two pharmacologically distinct receptor subtypes, B1 and B2 [6, 7]. The kinin B1 receptors (B1Rs) are normally absent or weakly expressed but are strongly induced following tissue injury or exposure to pro-inflammatory agents such as cytokines and toxins, while the kinin B2 receptors (B2Rs) are expressed constitutively [6, 7]. Kinin B2 receptors are activated by the intact kinins, bradykinin, and kallidin. Conversely, Kinin B1 receptors are activated preferentially by the carboxypeptidase metabolites of the kinins, des-Arg9-bradykinin (DBK) and des- Arg10-kallidin [6, 7]. Both B1R and B2R were detected in both normal human livers and HSCs, and were up-regulated in human fibrotic livers and activated HSCs [8]. B2R activated by bradykinin has been shown to attenuate liver damage and fibrosis development in a rat model of chronic liver injury [8]. By contrast, B1Rs are involved in diverse pathological processes, including inflammation, platelet activation, smooth muscle contraction, increased vascular permeability, oedema, pain, cytokine and chemokine release, cell proliferation, and tissue remodelling, responses that are key components of liver fibrosis [6, 7, 9–13]. Furthermore, in contrast to the B2 receptor mediated relaxation to bradykinin, B1Rs are induced in the rat portal vein and stimulation triggers vasoconstriction via the cyclooxygenase-2 (COX-2) pathway [14].

We hypothesized that B1R signalling may contribute to fibrotic pathways and PH in CLD. Small molecular non-peptide orally active antagonists of B1 receptors are desired for clinical drug development for a variety of inflammatory diseases. This study examined the effect of BI 113823, a small molecular non-peptide orally active inhibitor of B1Rs, in hepatic fibrosis and PH induced by carbon tetrachloride (CCl_4_) or bile duct ligation (BDL) [15–18]. The mechanisms underlying the protection afforded by B1R inhibition were also examined using human peripheral blood monocytes and neutrophils, as well as LX2 human HSCs (hHSCs).

## Methods

### Animals

Animal studies were approved by the Institutional Animal Care and Use Committee at Jeonbuk National University and complied with the Animal Welfare Act. In total, 96 male Balbc mice were used at 8–10 weeks and weighing 25–30 g. Mice were housed under controlled light/dark conditions and fed a standard diet with water ad libitum. All animals were observed daily for general health, and all invasive procedures were performed under aseptic conditions.

### Carbon tetrachloride (CCl_4_) induced liver fibrosis

Study design for CCl_4_ induced liver fibrosis model is shown in Additional file 1: Fig. S1A. Mice were randomly assigned to three study groups: (1) sham control, n = 12; (2) CCl_4_ + vehicle, n = 22; (3) CCl_4_ + BI 113823, n = 20. Liver fibrosis was induced by intraperitoneal administration of CCl_4_ (1 ml/kg/body weight dissolved in olive oil [1:3]) twice a week for 6 weeks [19]. Sham control mice received only olive oil injection. Mice received vehicle (0.1% Natrosol, p.o.) and BI 113823 (50 mg/kg, p.o., a gift from Boehringer Ingelheim Pharma KG, Biberach, Germany) daily for 6 weeks.

### Bile duct ligation (BDL) induced liver fibrosis

Study design for the BDL induced liver fibrosis model is shown in Additional file 1: Fig. S1B. Mice were anesthetized with ketamine (100 mg kg^−1^, i.m.) plus xylazine (10 mg kg^−1^, i.m.). A 2 cm abdominal midline incision was made and the common bile duct was located and tightly ligated with 4–0 silk suture [19]. Sham control mice underwent identical laparotomy without BDL (n = 12). Mice received daily treatment of vehicle (0.1% Natrosol, p.o., n = 18) and BI 113823 (50 mg/kg, p.o., n = 12) for 3 weeks.

### Portal vein pressure measurements

At the end of study protocol, mice were anesthetized as described above, an abdominal midline incision was made, and the portal vein was cannulated through an ileocolic vein with a 24-gauge catheter which was connected to a pressure transducer [20]. Portal vein pressure was recorded using a Powerlab data acquisition system (ADInstruments Inc., CO). At the end of the experiment, liver tissue was collected. Tissue samples were snap frozen in liquid nitrogen or fixed in buffered formalin for histopathological examination.

### Histopathological examination

Liver tissue specimens were fixed in 10% formalin and stained with haematoxylin and eosin (HE) and picrosirius red. Slides were examined by light microscopy for morphological alterations and collagen accumulation in a blinded fashion. Fibrosis and inflammatory cell accumulation were depicted through immunohistochemical staining for α-SMA/B1R, CD68 and neutrophil elastase. Total collagen content was assessed by hydroxyproline assay (BioVision kit), and inflammatory responses and profibrogenic mediators assessed by western blot and reverse transcription polymerase chain reaction (RT-PCR). Detailed methods are shown in Additional file 1.

### Human peripheral blood immune cell assay

Immune cell migration assays were carried out on monocytes and neutrophils treated with tumour necrosis factor (TNF)-α with or without BI 113823. Migrating cells were counted after 12 h by haemocytometer. Human peripheral monocytes were treated with lipopolysaccharides (LPS) with or without BI 113823 and TNF-α levels were measured with immunoassay kits (PreproTech, Rocky Hill, NJ, USA). Myeloperoxidase (MPO) assay was carried out on human peripheral neutrophils treated with LPS with or without BI 113823, and MPO release measured at 460 nm. Monocyte and neutrophil activation was determined by flow cytometry measuring upregulation of CD11b and CD18 on FACSCalibur apparatus.

### LX2 hHSC assays

Des-Arg9-bradykinin (DBK), a carboxypeptidase metabolite of the kinins, preferentially activates the kinin B1 receptors [7]. In isolated pig coronary arteries exposed to LPS, DBK caused induction of kinin B1 receptor-dependent, endothelium-independent contractions [17]. The changes in HSC cell proliferation and migration were measured in the presence of selective B1R agonist (DBK) and antagonist (BI 113823) in this study.

LX2 HSCs, kindly provided by Prof. Bumseok Kim, were grown in Dulbecco's modified Eagle's medium (DMEM) supplemented with 10% fetal bovine serum (FBS) and antibiotics. LX2 cell proliferation was determined by BrdU Cell Proliferation Assay Kit (BioVision) after incubation with or without different concentrations of des-Arg9-bradykinin (DBK) and BI 113823. Migration of HSCs was measured after incubation with 0.5% FBS containing medium with different concentrations of DBK and BI 113823 after 24 h. Cytoselect 48-well cell concentration assay (cell biolabs) was used to evaluate BI 113823’s effect on transforming growth factor (TGF)-β mediated HSC contraction. Photographs were made with a digital camera at 0, 24, and 48 h. The size of the gels were digitally measured and normalized with their respective well size using Image J software. HSCs were treated with BI 113823 in medium containing 1% FBS and analysed by flow cytometry to detect apoptosis (FITC Annexin V Apoptosis Detection Kit with PI, Biolegend). Cell cycle analysis was carried out by incubating starved HSCs with medium containing 1% FBS with or without DBK and BI 113823, and analysed using FxcyclePI/RNase staining kit. HSC transwell migration assay was performed on LX2 cells using medium with 1% FBS with or without BI 113823. Migrating cells were counted after 12 h by haemocytometer. HSCs were treated with TGF-β and BI 113823 for 24 h before overnight incubation with α-SMA Ab and visualization using rhodamine fluorescence probe-labelled secondary Ab. A western blot was carried out according to standard protocols using primary and secondary Abs as described in Additional file 1: Table S2.

### Statistical analysis

The results are presented as the mean ± SEM, unless otherwise specified. A two-group comparison was performed using unpaired Student’s t-test, while a multiple-group comparison was performed by analysis of variance for repeated measures followed by Tukey’s multiple comparison test, or Bonferroni test. Survival estimates were made by Kaplan–Meier analysis. Survival curves were analyzed by the nonparametric Mantel-Cox test.

## Results

### BI 113823 reduced CCl_4_-induced liver fibrosis and PH, and improves survival in mice

**Fig. 1:**
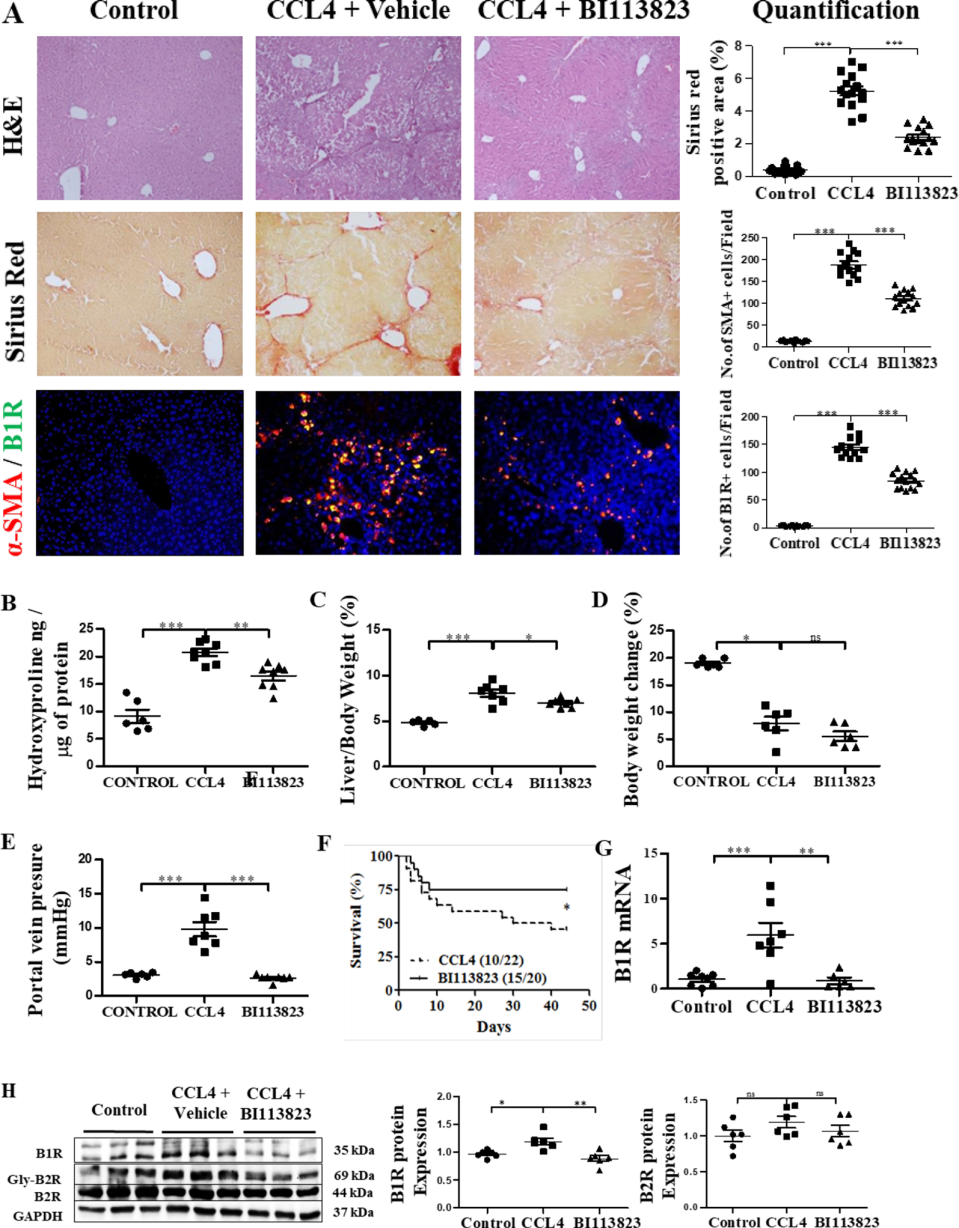
BI 113823 treatment reduced CCl_4_-induced liver fibrosis, PH and improved survival in mice. **A** Liver HE staining, sirius red staining, α-SMA (red) and B1 receptor (green) IFC stain and their quantification. **B** Hydroxyproline assay for total collagen in liver; **C** liver:bodyweight ratio; **D** bodyweight changes; **E** changes in portal vein pressure; and **F** survival rate. **G** The expression of B1 receptor mRNA in CCl_4_ mice livers; **H** Western blot for the protein expression of B1R, Gly-B2R, B2R, GAPDH in CCl_4_ mice livers. Values are mean ± SEM, n = 7–8. *p* values indicated in panels, *ns*. not significant and significant as *p < 0.05; **p < 0.01; ***p < 0.001. **A**–**E**, **G**–**H** One-way ANOVA and Tukey’s multiple comparison test were performed. **F** Survival curves were analyzed by the nonparametric Mantel-Cox test

Liver fibrosis in mice was evaluated by HE and sirius red staining, and hydroxyproline assay at 6 weeks after the initial CCl_4_ challenge. HE staining of liver sections from CCl_4_-treated mice showed prominent hepatic steatosis, necrosis, fibrotic septa formation, disruption of tissue architecture, and inflammatory cell infiltration (Fig. 1A). Liver sections from BI 113823-treated mice showed relatively normal architecture (Fig. 1A). Treatment with BI 113823 significantly reduced CCl_4_-induced liver fibrosis, as demonstrated by reduction in sirius red staining for collagen and reduction of hydroxyproline content in liver (Fig. 1A and B). HSC activation and differentiation, as assessed by immunofluorescence staining for α-SMA, showed marked increase in CCl_4_-treated mice, and this was correlated with marked induction of B1R expression in CCl_4_-induced fibrotic liver (Fig. 1A). The expression of α-SMA and B1Rs in liver tissue was significantly reduced in mice treated with BI 113823 (Fig. 1A, G and H). B2R expression was not changed (Fig. 1H). Immunofluorescence double staining results showed that cells expressing the B1Rs co-localized with those expressing α-SMA (Fig. 1A). These findings indicate B1R signalling mediates CCl_4_-induced liver fibrosis via activation of HSCs.

Six weeks after the initial CCl_4_ challenge, bodyweight growth was slightly lower compared to the sham control (not significantly different, Fig. 1D). However, liver/body weight ratio and portal vein pressure were significantly increased in CCl_4_-challenged mice, compared to the sham control (Fig. 1C). CCl_4_-induced liver fibrosis and PH were associated with high mortality rate in those mice (12/22 death, Fig. 1F). In contrast, mice that received BI 113823 showed significantly lower liver/body weight ratio, lower portal vein pressure and, most importantly, improved survival (Fig. 1C, E and F).

### BI 113823 reduced BDL-induced liver fibrosis and PH, and improved survival in mice

**Fig. 2:**
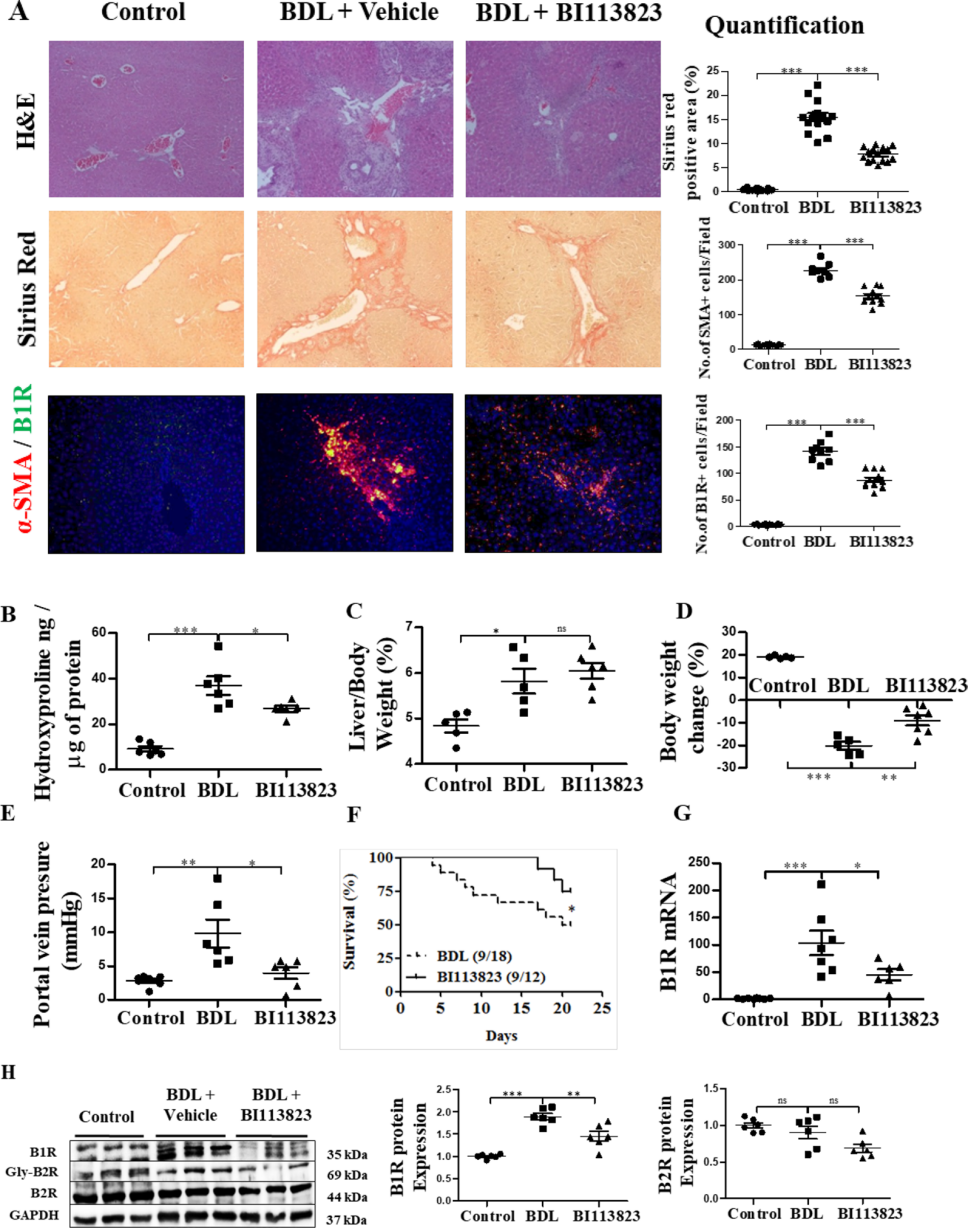
BI 113823 treatment reduced BDL-induced liver fibrosis and PH, and improved survival in mice. **A** Liver HE staining, sirius red staining, α-SMA (red) and B1 receptor (green) IFC stain and their quantification. **B** Hydroxyproline assay for total collagen in liver; **C** liver:bodyweight ratio; **D** bodyweight changes; **E** changes in portal vein pressure; and **F** survival rate; **G** The expression of B1 receptor mRNA in BDL mice livers; **H** Western blot for the protein expression of B1R, Gly-B2R, B2R, GAPDH in BDL mice livers. Values are mean ± SEM, n = 7–8. *p* values indicated in panels, *ns*. not significant and significant as *p < 0.05; **p < 0.01; ***p < 0.001. **A**–**E**, **G**–**H** One-way ANOVA and Tukey’s multiple comparison test were performed. **F** Survival curves were analyzed by the nonparametric Mantel-Cox test

In our next model, liver fibrosis in mice was evaluated 3 weeks after BDL. Liver sections from BDL mice showed marked hepatic steatosis, necrosis, inflammatory cell infiltration and fibrotic septa formation, as well as extensive collagen deposition as accessed by HE and sirius red staining, and hydroxyproline assay (Fig. 2A and B). Similarly, liver fibrosis in BDL mice was significantly reduced in mice treated with BI 113823, compared to vehicle controls (Fig. 2A). Liver fibrosis in BDL mice was also associated with marked increase of α-SMA expression, as well as co-localized induction of B1Rs. BI 113823 reduced the expression of α-SMA and B1Rs in BDL mice (Fig. 2A, G and H). These findings further support the hypothesis that B1R signalling mediates chronic liver fibrosis via activation of HSCs.

Three weeks after BDL, liver/body weight ratio was not significantly different among groups (Fig. 2C) and body weight was significantly decreased in vehicle-treated BDL mice, compared to sham control (Fig. 2D). However, the decrease of body weight after BDL was less in BI 113823-treated mice (Fig. 2D). BDL resulted in severe PH, and BI 113823 significantly decreased portal vein pressure (Fig. 2E) and improved survival (Fig. 2F).

Altogether, these data demonstrate that, in experimental models of CLD, B1R inhibition with BI 113823 attenuates hepatic fibrosis formation, PH and, most importantly improves survival.

### BI 113823 reduced the expression of profibrogenic mediators in liver after chronic CCl_4_ challenge and BDL

**Fig. 3:**
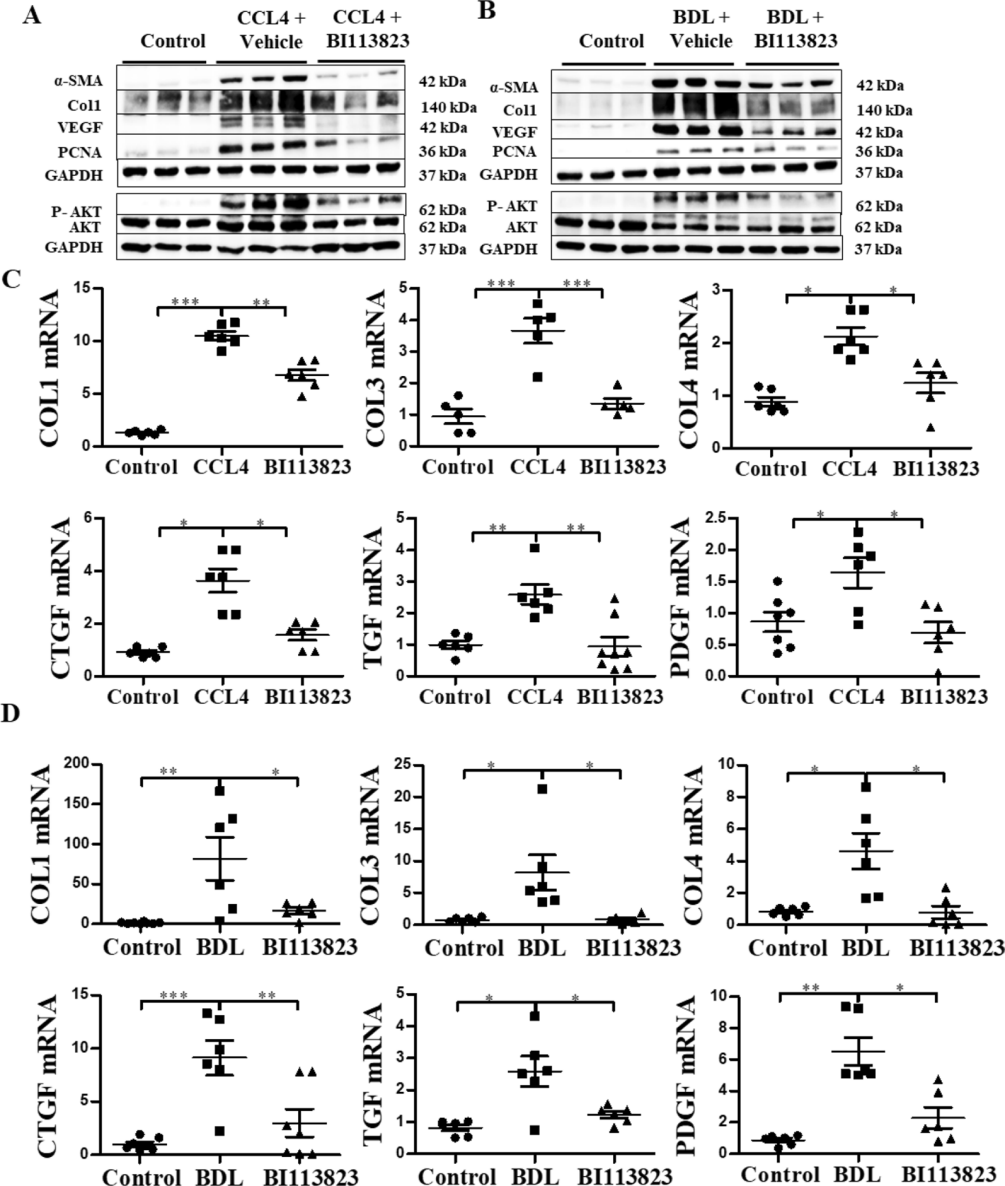
BI 113823 treatment reduced the expression of fibrosis mediators in CCl_4_ and BDL mice livers. **A**, **B** Western blot for the protein expression of α-SMA, Col1, VEGF, PCNA, GAPDH and P-AKT, AKT, GAPDH in **A** CCl_4_ and **B** BDL mice livers. **C**, **D** The expression of Col1, Col2, Col4, TGF, CTGF and PDGF mRNA in **C** CCl_4_ and **D** BDL mice livers. Values are mean ± SEM, n = 7–8. *p* values indicated in panels, *ns*. not significant and significant as *p < 0.05; **p < 0.01; ***p < 0.001. One-way ANOVA and Tukey’s multiple comparison test were performed

We next determined the expression of profibrotic mediators in CCl_4_- and BDL-induced liver fibrosis. Western blot analysis showed an extensive increase in the expression of profibrotic mediators α-SMA, collagen I, VEGF and proliferating cell nuclear antigen (PCNA) in CCl_4_- and BDL-induced liver fibrosis (Fig. 3A and B, Additional file 1: Fig. S2A and B). Compared to vehicle-treated mice, expression of α-SMA, collagen I, VEGF and PCNA were all significantly reduced in mice treated with BI 113823 (Fig. 3A and B). Furthermore, hepatic expression of phosphorylated Akt protein was significantly increased in CCl_4_- and BDL-induced liver fibrosis (Fig. 3A and B), and BI 113823 reduced this expression (Fig. 3A and B, Additional file 1: Fig. S2A and S2B).

RT-PCR showed that hepatic mRNA expressions of growth factors PDGF, TGF and CTGF, as well as ECM molecules Col-1, Col-3 and Col-4 increased significantly in CCl_4_- and BDL-induced liver fibrosis, and all were reduced in mice treated with BI 113823 (Fig. 3C and D). Together, these findings further demonstrate that BI 113823 attenuates hepatic fibrosis via inhibition of HSC activation and proliferation, downregulates the expression of fibrogenic mediators via inhibition of the Akt signalling pathway.

### BI 113823 reduced inflammatory responses in CCl_4_- and BDL-induced liver fibrosis

**Fig. 4:**
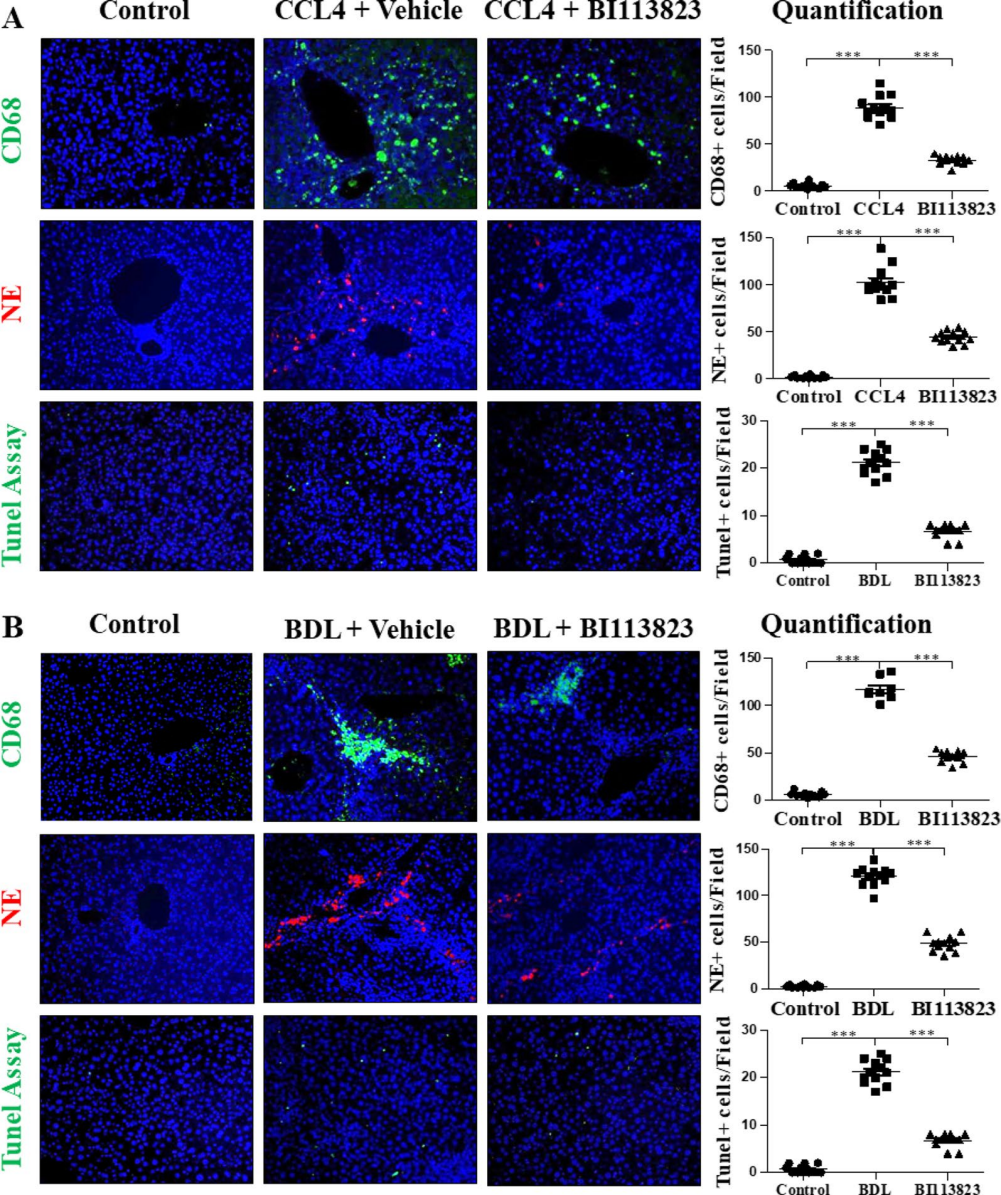
BI 113823 treatment reduced liver macrophage and neutrophil accumulation, and liver apoptosis in CCl_4_- and BDL-induced liver fibrosis. IFC stain for macrophages and neutrophils, staining for apoptosis, and their quantifications in **A** CCl_4_ and **B** BDL mice liver. Values are mean ± SEM, n = 7–8. *p* values indicated in panels, *ns*. not significant and Significant as *p < 0.05; **p < 0.01; ***p < 0.001. One-way ANOVA and Tukey’s multiple comparison test were performed

Chronic CCl_4_ challenge and BDL resulted in marked hepatic inflammatory cell infiltration in mice (Fig. 4A and B). Immunohistochemical staining of liver sections showed a marked increase of CD68 positive macrophages and neutrophils (positive for neutrophil elastase) in both CCl_4_- and BDL-induced liver fibrosis (Fig. 4A and B). BI 113823 reduced hepatic inflammatory cell infiltration of macrophages and neutrophils, and hepatocyte apoptosis (Fig. 4A and B).

**Fig. 5:**
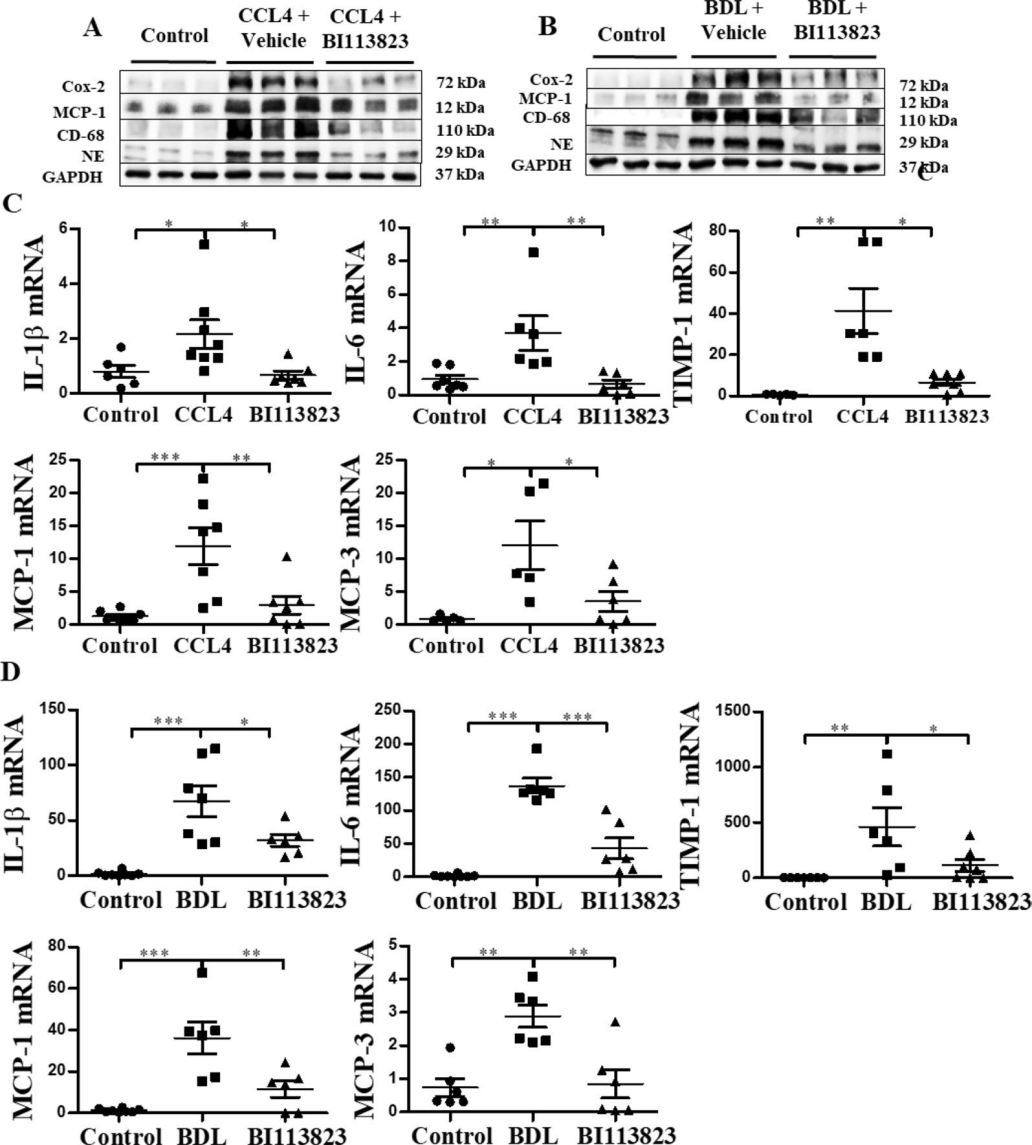
BI 113823 treatment reduced the expression of inflammatory mediators in CCl_4_ and BDL mice livers. **A**, **B** Western blot for the protein expression of COX-2, MCP-1, CD68, neutrophil elastase, GAPDH in **A** CCl_4_ and **B** BDL mice livers. **C**, **D** The expression of IL-1, IL-6, MCP-1, MCP-3 and TIMP-1 mRNA in **C** CCl_4_ and **D** BDL mice livers. Values are mean ± SEM, n = 7–8. *p* values indicated in panels, *ns*. not significant and Significant as *p < 0.05; **p < 0.01; ***p < 0.001. One-way ANOVA and Tukey’s multiple comparison test were performed

To provide further evidence that B1Rs mediate inflammatory cell infiltration and inflammatory mediator production in CCl_4_- and BDL-induced liver fibrosis, we assessed protein expression of inflammatory cell markers CD68 and neutrophil elastase, and chemoattractant markers MCP-1 and COX-2 in liver tissues. Western blot of liver lysates showed that inflammatory molecules, COX-2, MCP-1, CD68, and neutrophil elastase were strongly increased in CCl_4_- and BDL-induced liver fibrosis, compared to sham control mice (Fig. 5A, B, Additional file 1: Fig. S3A and B). BI 113823 significantly reduced hepatic expression of these inflammatory mediators (Fig. 5A, B, Additional file 1: Fig. S3A and B).

RT-PCR analyses supported these observations by showing that mRNAs of inflammatory mediators, IL-1β, IL-6, MCP-1, MCP-3 and TIMP-1 increased in CCl_4_- and BDL-induced liver fibrosis, and decreased in BI 113823-treated mice (Fig. 5C and D). These findings indicate that B1R-mediated inflammatory responses are highly implicated in the pathogenesis of liver fibrosis.

### BI 113823 reduced human inflammatory cell migration and activation

**Fig. 6:**
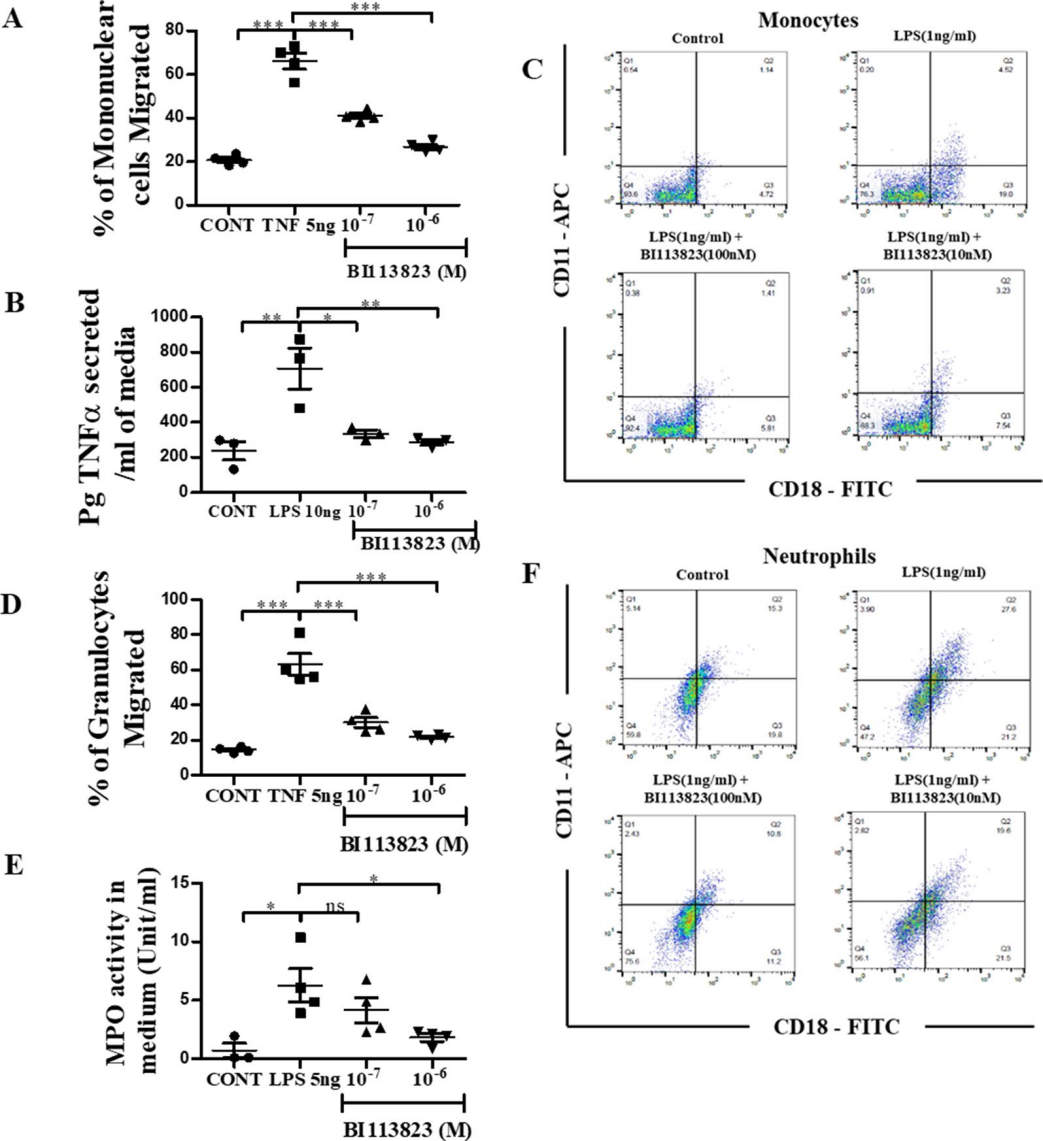
BI 113823 treatment reduced human peripheral blood monocyte and neutrophil activation, migration and cytokine release. **A** TNF-α-induced migration in monocytes. **B** LPS-induced TNF-ɑ secretion in monocytes. **C** LPS-induced CD11/CD18 integrin’s expression FACS dot plots in monocytes. **D** TNF-α-induced neutrophils migration. **E** LPS-induced MPO activity in neutrophils. **F** LPS-induced CD11/CD18 integrin’s expression FACS dot plots in neutrophils. Values are mean ± SEM, n = 3–4. *p < 0.05; **p < 0.01; ***p < 0.001. *p* values indicated in panels, *ns*. not significant and Significant as *p < 0.05; **p < 0.01; ***p < 0.001. One-way ANOVA and Tukey’s multiple comparison test (**A**, **B**, **D**, **E**) were performed

We next determined the effect of B1R-mediated inflammatory responses and activation in human monocytes and neutrophils. BI 113823 inhibited TNF-α-induced monocyte and neutrophil transmigration (Fig. 6A and D), reduced LPS-induced TNF-α production in monocytes and reduced LPS-stimulated MPO activity in neutrophils (Fig. 6B and E). LPS treatment resulted in a significant increase in activation of human monocytes and neutrophils, as evidenced by an increase in cell surface molecule CD11 and CD18 expression (Fig. 6C and F, Additional file 1: Fig. S4A and S4B). LPS-induced monocyte and neutrophil activation were inhibited by BI 113823 treatment (Fig. 6C and F, Additional file 1: Fig. S4A and B).

### BI 113823 reduced hHSC activation, contraction, migration and fibrosis protein expression

**Fig. 7:**
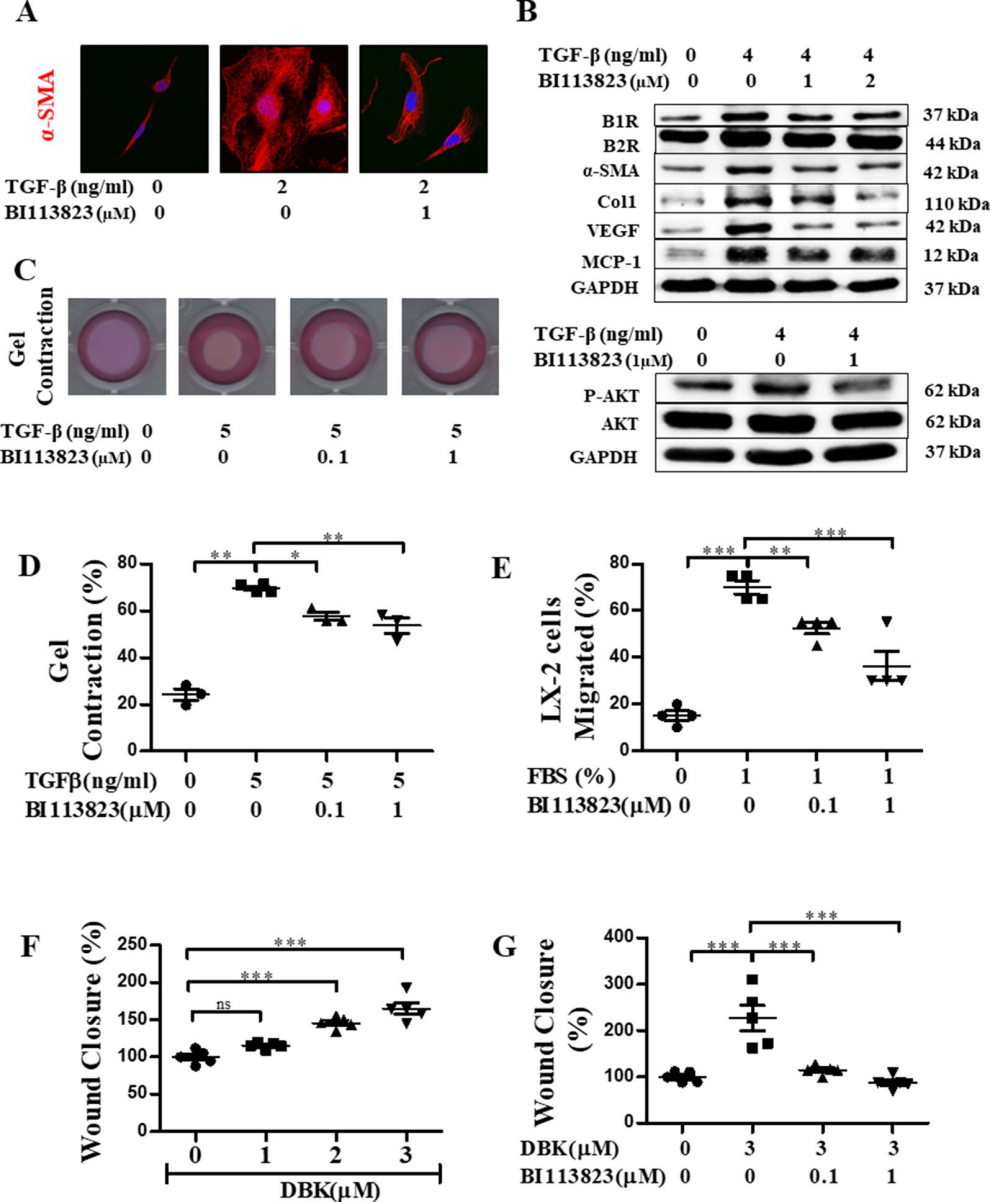
BI 113823 treatment significantly reduced LX-2 hHSC activation, proliferation, migration and contraction. **A** α-SMA expression. **B** Western blot for the protein expression of B1R, B2R, αSMA, Col1, VEGF, MCP-1 and phosphorylation of AKT. **C**, **D** Gel contraction and quantification. **E** Cell migration. **F**, **G** DBK-induced wound closure. Data are representative of three independent experiments, with n = 3. *p* values indicated in panels, *ns*. not significant and significant as *p < 0.05; **p < 0.01; ***p < 0.001. One-way ANOVA and Tukey’s multiple comparison test were performed

TGF-β is a major profibrogenic cytokine responsible for initiation and perpetuation of HSC activation. In this study, TGF-β stimulated strong intracellular expression of α-SMA in LX2 hHSCs, indicating HSC activation switching from a quiescent state to a myofibroblast phenotype**,** which was inhibited by BI 113823 (Fig. 7A). In collagen gel contraction assays, BI 113823 reduced TGF-β-stimulated hHSC contractility measured at 48 h by final gel area (Fig. 7C and D). Next, we determined the downstream effect of myofibroblast transdifferentiation, specifically fibrosis molecules production. Western blot analysis showed that TGF-β stimulated a significant increase in the expression of profibrogenic proteins α-SMA, Col-1, VEGF and MCP-1, as well as the expression of B1Rs and B2Rs in LX-2 hHSCs. Treatment of hHSCs with BI 113823 reduced the expression of profibrotic proteins and B1Rs (Fig. 7B). Furthermore, BI 113823 treatment inhibited TGF-β stimulated phosphorylation of Akt in LX-2 hHSCs (Fig. 7B).

HSCs can migrate to the sites of tissue injury during fibrogenesis and differentiate into contractile myofibroblasts that promote liver stiffness and PH. We further examined the effects of BI 113823 using migration (Fig. 7E) and wound-healing assays (Fig. 7F and G), where the selective B1R agonist [21] DBK stimulated hHSCs migration in a dose-dependent manner (Fig. 7F). BI 113823 treatment significantly inhibited FBS- and DBK-stimulated migration in LX-2 hHSCs (Fig. 7E–G).

### BI 113823 inhibits DBK stimulated HSC proliferation, arrests cell cycle from G1 to S phase transition, and blocks of PI3K/AKT signalling pathway

**Fig. 8:**
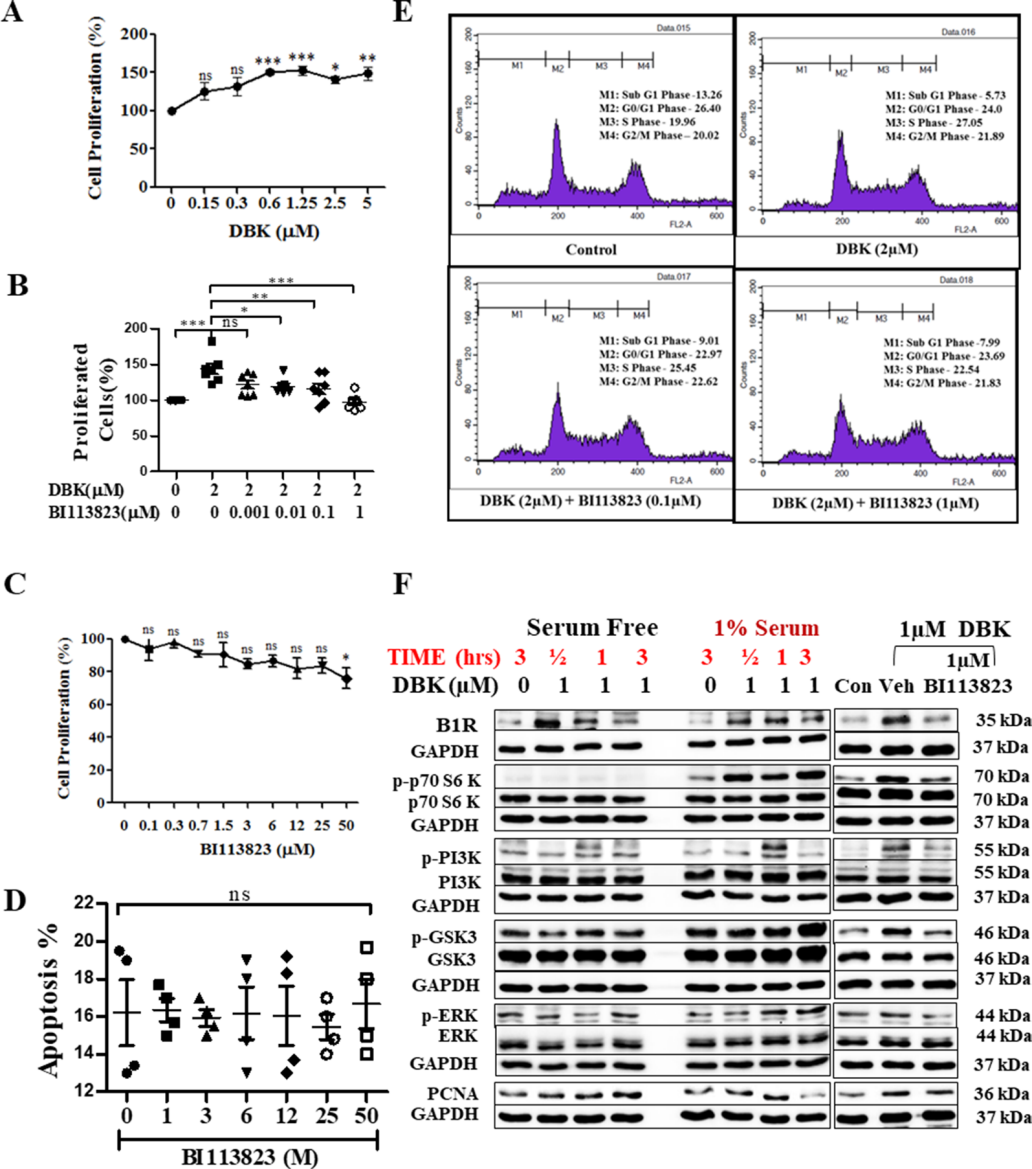
BI 113823 inhibited DBK-induced LX-2 hHSC proliferation, cell cycle regulation and inhibition of the PI3K signalling pathway. **A** DBK-induced LX-2 human HSC proliferation. **B** BI 113823 inhibited DBK-induced cell proliferation. **C** BI 113823 on normal cell proliferation. **D** Apoptosis. **E** BI 113823 on DBK-induced cell cycle progression. **F** Protein expression of B1R, PCNA and phosphorylation of pP70s6 kinase, pPI3K, pGSK3 and pERK. Data are representative of three independent experiments, with n = 3. *p* values indicated in panels, *ns*. not significant and significant as *p < 0.05; **p < 0.01; ***p < 0.001. One-way ANOVA and Tukey’s multiple comparison test were performed

In this study, we found that the selective B1R agonist DBK enhances the proliferation of hHSCs (Fig. 8A). BI 113823 dose-dependently inhibited DBK-stimulated hHSC proliferation (Fig. 8B) but did not affect non-DBK-stimulated hHSC growth or apoptosis (Fig. 8C and D). Flow cytometry analysis showed that DBK (2 µM) stimulated the entry of hHSCs into S phase of the cell cycle. At 1 µM, BI 113823 reduced DBK-stimulated G1 to S phase cell cycle transition by 64% (Fig. 8E, Additional file 1: Fig. S5A). Taken together, the present study provides direct evidence that DBK induces hHSC proliferation and migration via activation of B1Rs.

Western blot analysis showed that B1Rs were weakly expressed in serum-free quiescent or growing hHSCs, but were strongly upregulated by DBK (Fig. 8F, Additional file 1: Fig. S5B). DBK also stimulated phosphorylation of P70S6 kinase, PI3K, GSK3, ERK and increased the expression of PCNA in HSCs (Fig. 8F, Additional file 1: Fig. S5B). Interestingly, phosphorylation of P70S6 kinase was not detected in serum-free quiescent HSCs with or without DBK, but DBK stimulated strong phosphorylation of P70S6 kinase in HSCs with 1% FBS. BI 113823 treatment inhibited phosphorylation of P70S6 kinase, PI3K, GSK3 and ERK, blocked B1R induction, and reduced expression of PCNA (Fig. 8F, Additional file 1: Fig. S5B). Together, these findings demonstrate that DBK-stimulated B1R signalling mediates HSC proliferation and migration via activation of the PI3K/Akt pathway.

## Discussion

The novel finding of the present study is that B1R blockade with BI 113823 effectively prevented the development of liver fibrosis in two well-established experimental models of CLD. In mice with CCl_4_- and BDL-induced CLD, BI 113823 reduced inflammatory cell infiltration, liver fibrosis formation, PH, and, most importantly, improved survival. Moreover, our study also demonstrates that B1R activation regulates leukocyte infiltration; proinflammatory cytokine production, HSC activation, proliferation, migration and contraction, expression of profibrogenic mediators, as well as ERK and PI3K/Akt signalling pathways**.** The inhibition of B1R-associated signalling pathways with BI 113823 demonstrates the importance of DBK-stimulated B1R signalling in the pathogenesis of liver fibrosis and PH (Additional file 1: Fig. S6).

### B1Rs in hepatic inflammation

Hepatocyte damage results in the recruitment of inflammatory cells, and upregulation of inflammatory and fibrogenetic mediators, including various cytokines, chemokines that activate HSCs and drive liver fibrosis, and activation of Kupffer cells [2, 21]. Kinins mediate liver inflammatory injury and fibrosis through constitutively expressed B2 receptors and through induction of B1 receptors [6, 7]. Inflammatory cells are also a major source of tissue kallikrein, which increases kinin production [22]. Increased blood level of tissue kallikrein and reduced hepatic clearance of plasma kallikrein was associated with liver cirrhosis patients and cirrhotic rats [23, 24]. B1Rs are induced by stimulation of lipopolysaccharides, IL-1β and TNF-α, by tissue injury and exposure to kinins (the receptor agonists) via activation of NF-ĸB signaling pathway [6, 7, 15–18, 25]. B1 receptor induction elicits persistent inflammatory responses and its up-regulation is further boosted by ligand binding [17, 26]. Therefore, kinin B1 receptors may be important in maintaining chronic inflammatory processes, such as chronic liver injury [8].

The present study discovered that DBK stimulates LX2 human HSC proliferation. In addition to induction of B1Rs by inflammatory mediator TGF-β, we also show that in HSCs, B1Rs are strongly induced by the receptor agonist DBK. This is consistent with a previous report that B1Rs can be induced by exposure to inflammatory mediators and its receptor agonists [25]. Using human peripheral blood inflammatory cells, we showed that B1Rs mediate human monocytes and neutrophil activation, migration and cytokine production. These findings suggest that blocking B1Rs to control inflammatory responses would be protective for liver fibrosis. In this study, CCl_4_- and BDL-induced liver fibrosis was associated with a marked increase of macrophage and neutrophil accumulation and strong upregulation of B1Rs in liver tissue. B1R blockade with BI 113823 inhibited the accumulation of macrophages and neutrophils in both CCl_4_ and BDL fibrosis livers, and reduced the expression of inflammatory mediators, B1Rs, COX-2, IL-1β, IL-6, MCP-1, MCP-3 and TIMP-1 in liver tissue. Taken together, our results demonstrated that blocking inflammatory responses with BI 113823 contributed to reduced chronic liver fibrosis in mice.

### B1Rs in hepatic fibrosis

Kinin B1 receptors is strongly expressed in regenerating and cirrhotic livers [8, 27–29]. In human lung myofibroblasts, B1R activation by DBK triggers synthesis and secretion of matrix metalloproteases, type I collagen synthesis and the expression of connective tissue growth factor [29, 30]. B1R blockade inhibits vascular cell proliferation and vascular remodelling, and attenuates cardiac fibrosis and hypertrophy [13, 31]. Mice lacking B1Rs display an improvement on leptin and insulin sensitivity and are protected from non-alcoholic fatty liver disease (NAFLD) after a high-fat diet treatment [32]. Collectively, these observations suggest that B1Rs may play an important role in hepatic fibrosis.

In the present study, B1R mRNA and protein were weakly expressed in normal mice liver, but were strongly increased in CCl_4_ and BDL fibrosis mice. The induction of B1Rs was correlated with severe hepatic fibrosis in CCl_4_ and BDL mice, with excessive collagen deposition and fibrotic molecules expression. B1R blockade with BI 113823 inhibited HSC activation, fibrotic mediator expression and collagen production, and reduced mouse liver fibrosis induced by treatment with CCl_4_ and BDL. These findings support the hypothesis that B1 receptors may be important in the pathogenesis of liver fibrosis.

HSCs are recognised as the primary cellular source of matrix components in patients with CLD [33]. When activated, HSCs proliferate and transdifferentiate into myofibroblasts, and these cells produce fibrogenic molecules in the hepatic ECM [1–5]. Pro-inflammatory and pro-fibrotic cytokines such as TGF-β and PDGF are recognised as common mediators of HSC activation and proliferation [34]. In the present study, we discovered that the selective B1 agonist DBK is a strong mediator of HSC activation and proliferation by stimulation of the B1R pathway. B1Rs were weakly expressed in quiescent hHSCs, but were strongly induced in response to TGF-β and DBK. DBK enhanced HSC proliferation. Interestingly, B1Rs blockade with BI 113823 inhibited DBK-stimulated HSC proliferation, but did not affect the proliferation and apoptosis in the normal culture of HSCs. BI 113823 blocked DBK-stimulated HSC proliferation by preventing G1 to S phase cell cycle transition. The activation of HSCs can induce α-SMA-positive myofibroblast transition, strengthen their proliferation and increase collagen synthesis [33]. In this study, B1Rs blockade with BI 113823 inhibited TGF-β and DBK stimulated HSC activation, migration and reduced the expression of fibrotic molecules such as α-SMA, collagen-1, VEGF and MCP-1. Our findings demonstrate that DBK is a strong activator of HSC and the DBK-stimulated B1R signalling pathway, which regulates HSC activation, proliferation and migration, and contributes to fibrogenesis in chronic liver injury.

The PI3K signalling pathway promotes cell proliferation and collagen synthesis in HSCs, and inhibition of PI3K signalling in activated HSCs and fibrosis models inhibit the expression of collagen and attenuates the development of liver fibrosis [33, 35]. In the present study, the increase in HSC activation, cell proliferation and collagen production in CCl_4_- and BDL-induced fibrotic liver was correlated with strong phosphorylation of Akt, and B1R blockade with BI 113823, inhibited the phosphorylation of Akt, and reduced liver fibrosis. We also discovered that DBK induced a marked increase of expression of B1Rs in quiescent and growing hHSCs. The induction of B1Rs in hHSCs was correlated with an increase of PCNA and the phosphorylation of P70S6 kinase, PI3K, GSK3 and ERK. Treatment with BI 113823 inhibited DBK-stimulated phosphorylation of P70S6 kinase, PI3K, GSK3 and ERK, and inhibited DBK-induced HSC proliferation and migration. Taken together, our results demonstrated that the inhibition of HSC activation, proliferation and fibrosis formation by BI 113823 involves blocking PI3K/Akt signalling pathways both in vitro and in vivo**.**

### B1Rs in PH

In portal hypertension, damaged parenchymal and non- parenchymal cells contribute to the increase of intrahepatic vascular resistance (IHVR) in cirrhosis through two major mechanisms: a profound alteration in liver architecture and a pathological increase in the hepatic vascular tone [36–38]. Hepatic fibrosis and sinusoidal capillarization are major contributors to these structural changes and IHVR in chronic liver diseases [36]. IHVR also attributed to deregulated contractile elements, such as endothelin 1 (ET1) and thromboxane A2 (TXA2), and are hyporesponsive to vasodilators, such as nitric oxide (NO) and prostacyclin [36, 37, 39–41]. B1Rs are induced in rat portal veins and pig coronary arteries and stimulation triggers vasoconstriction via the cyclooxygenase-2 (COX-2) and TXA2 pathway [14, 17]. Elevated VEGF levels exacerbate portal hypertension by increasing nitric oxide production and angiogenesis.

In this study, B1R expression was strongly increased in hHSCs in response to TGF-β and DBK, and is also increased in the livers of CCl_4_ and BDL fibrosis mice. B1R blockade with BI 113823 inhibited hHSC contractility in collagen gel contraction assays, attenuated PH in CCl_4_ and BDL fibrosis mice. This reduction in portal vein pressure was associated with downregulation of VEGF and COX-2 expression in both mice models, as well as reduction of VEGF protein expression in LX-2 hHSC cells. These results provide novel insight into the role of B1R expression and the regulation of hepatic vascular tone in diseases.

## Conclusion

The present study discovers a novel mechanism that B1Rs mediate the pathogenesis of chronic liver fibrosis and PH. B1R antagonists merit consideration as a novel therapeutic approach for chronic liver fibrosis and PH.

## Supplementary Information

**Additional file 1.** **Figure S1.** Study schema for (A) Carbon tetrachloride (CCl_4_) induced liver fibrosis and (B) Bile duct ligation (BDL) induced liver fibrosis. **Figure S2.** Densitometric analysis of western blots for protein expression of αSMA, Col1, VEGF, PCNA & P-AKT / AKT in (A) CCl4 and (B) BDL mice liver. **Figure S3.** Densitometric analysis of western blots for protein expression of COX-2, MCP-1, CD-68, and NE in (A) CCl4 and (B) BDL mice liver. **Figure S4.** FACS dot plots quantification graphs of Fig. 6C, F. **Figure S5.** (A) Cell Cycle flowcytometric plots quantification graphs of Fig. 8E; (B) Densitometric analysis of western blots (Fig. 8F) for protein expression of B1R, PCNA and phosphorylation of pP70s6 kinase, pPI3K, pGSK3 and pERK in LX-2 cells treated with DBK and BI113823. **Figure S6.** Schematic illustration of kinin B1 receptors in the pathogenesis of liver fibrosis. **Table S1.** Materials used in PCR. **Table S2.** Materials, primary and secondary antibodies.

## Data Availability

The datasets used and/or analyzed during the current study are available from the corresponding author on reasonable request.

## References

[1] Khanam A, Saleeb PG, Kottilil S (2021). Pathophysiology and treatment options for hepatic fibrosis: can it be completely cured?. Cells.

[2] Roehlen N, Crouchet E, Baumert TF (2020). Liver fibrosis: mechanistic concepts and therapeutic perspectives. Cells.

[3] Ye F, Zhai M, Long J, Gong Y, Ren C, Zhang D, Lin X, Liu S (2022). The burden of liver cirrhosis in mortality: results from the global burden of disease study. Front Public Health.

[4] Brandon-Warner E, Benbow JH, Swet JH, Feilen NA, Culberson CR, McKillop IH (2018). Adeno-associated virus serotype 2 vector-mediated reintroduction of microRNA-19b attenuates hepatic fibrosis. Hum Gene Ther.

[5] Puche JE, Saiman Y, Friedman SL (2013). Hepatic stellate cells and liver fibrosis. Compr Physiol.

[6] Girolami JP, Bouby N, Richer-Giudicelli C, Alhenc-Gelas F (2021). Kinins and kinin receptors in cardiovascular and renal diseases. Pharmaceuticals.

[7] Sriramula S (2020). Kinin B1 receptor: a target for neuroinflammation in hypertension. Pharmacol Res.

[8] Sancho-Bru P, Bataller R, Fernandez-Varo G, Moreno M, Ramalho LN, Colmenero J, Marí M, Clària J, Jiménez W, Arroyo V, Brenner DA, Ginès P (2007). Bradykinin attenuates hepatocellular damage and fibrosis in rats with chronic liver injury. Gastroenterology.

[9] Tidjane N, Hachem A, Zaid Y, Merhi Y, Gaboury L, Girolami JP (2015). A primary role for kinin B1 receptor in inflammation, organ damage, and lethal thrombosis in a rat model of septic shock in diabetes. Eur J Inflamm.

[10] Raslan F, Schwarz T, Meuth SG, Austinat M, Bader M, Renné T (2010). Inhibition of bradykinin receptor B1 protects mice from focal brain injury by reducing blood-brain barrier leakage and inflammation. J Cereb Blood Flow Metab.

[11] Rampa DR, Murugesan P, Chao H, Feng H, Dai W, Pekcec A (2021). Reversal of pulmonary arterial hypertension and neointimal formation by Kinin B1 receptor blockade. Respir Res.

[12] Westermann D, Walther T, Savvatis K, Escher F, Sobirey M, Riad A (2009). Gene deletion of the kinin receptor B1 attenuates cardiac inflammation and fibrosis during the development of experimental diabetic cardiomyopathy. Diabetes.

[13] Murugesan P, Hildebrandt T, Bernlöhr C, Lee D, Khang G, Doods H (2015). Inhibition of kinin B1 receptors attenuates pulmonary hypertension and vascular remodeling. Hypertension.

[14] Basei FL, Cabrini DA, Figueiredo CP, Forner S, Hara DB, Nascimento AF (2012). Endothelium dependent expression and underlying mechanisms of des-Arg^9^-bradykinin-induced B_1_R-mediated vasoconstriction in rat portal vein. Peptides.

[15] Gurusamy M, Nasseri S, Lee H, Jung B, Lee D, Khang G (2016). Kinin B1 receptor antagonist BI 113823 reduces allergen-induced airway inflammation and mucus secretion in mice. Pharmacol Res.

[16] Murugesan P, Jung B, Lee D, Khang G, Doods H, Wu D (2016). Kinin B1 receptor inhibition with BI 113823 reduces inflammatory response, mitigates organ injury, and improves survival among rats with severe sepsis. J Infect Dis.

[17] More AS, Kim HM, Khang G, Hildebrandt T, Bernlöhr C, Doods H (2014). Des-Arg9-bradykinin causes kinin B1 receptor mediated endothelium-independent contractions in endotoxin-treated porcine coronary arteries. Pharmacol Res.

[18] Nasseri S, Gurusamy M, Jung B, Lee D, Khang G, Doods H, Wu D (2015). Kinin B1 receptor antagonist BI113823 reduces acute lung injury. Crit Care Med.

[19] Osawa Y, Oboki K, Imamura J, Kojika E, Hayashi Y, Hishima T, Saibara T, Shibasaki F, Kohara M, Kimura K (2015). Inhibition of cyclic adenosine monophosphate (cAMP)-response element-binding protein (CREB)-binding protein (CBP)/β-catenin reduces liver fibrosis in mice. EBioMedicine.

[20] Geerts AM, Vanheule E, Praet M, Van Vlierberghe H, De Vos M, Colle I (2008). Comparison of three research models of portal hypertension in mice: macroscopic, histological and portal pressure evaluation. Int J Exp Pathol.

[21] Sahin H, Trautwein C, Wasmuth HE (2010). Functional role of chemokines in liver disease models. Nat Rev Gastroenterol Hepatol.

[22] Lauredo IT, Forteza RM, Botvinnikova Y, Abraham WM (2004). Leukocytic cell sources of airway tissue kallikrein. Am J Physiol Lung Cell Mol Physiol.

[23] Yuki N, Kubo M, Noro Y, Hayashi N, Fusamoto H, Ito A (1991). Renal kinin and kallikrein excretion in cirrhotic patients. Scand J Gastroenterol.

[24] Nagaoka MR, Kouyoumdjian M, Borges DR (2003). Hepatic clearance of tissue-type plasminogen activator and plasma kallikrein in experimental liver fibrosis. Liver Int.

[25] Phagoo SB, Poole S, Leeb-Lundberg LM (1999). Autoregulation of bradykinin receptors: agonists in the presence of interleukin-1beta shift the repertoire of receptor subtypes from B2 to B1 in human lung fibroblasts. Mol Pharmacol.

[26] Nagaoka MR, Gomiero L, Teixeira FO, Agostino FG, Pouza JEP, Mimary P (2006). Is the expression of kinin B1 receptor related to intrahepatic vascular response?. Biochim Biophys Acta.

[27] Faussner A, Bathon JM, Proud D (1999). Comparison of the responses of B1 and B2 kinin receptors to agonist stimulation. Immunopharmacology.

[28] Kouyoumdjian M, Nagaoka MR, Borges DR (2005). Kallikrein-kinin system in hepatic experimental models. Peptides.

[29] Romero JR, Rivera A, Lança V, Bicho MD, Conlin PR, Ricupero DA (2005). Na+/Ca2+ exchanger activity modulates connective tissue growth factor mRNA expression in transforming growth factor beta1- and Des-Arg10-kallidin-stimulated myofibroblasts. J Biol Chem.

[30] Ricupero DA, Romero JR, Rishikof DC, Goldstein RH (2000). Des-Arg(10)-kallidin engagement of the B1 receptor stimulates type I collagen synthesis via stabilization of connective tissue growth factor mRNA. J Biol Chem.

[31] Lin X, Bernloehr C, Hildebrandt T, Stadler FJ, Doods H, Wu D (2016). Kinin B1 receptor blockade and ACE inhibition attenuate cardiac postinfarction remodeling and heart failure in rats. Toxicol Appl Pharmacol.

[32] Fonseca RG, Sales VM, Ropelle E, Barros CC, Oyama L, Ihara SS (2013). Lack of kinin B_1_ receptor potentiates leptin action in the liver. J Mol Med (Berl).

[33] Son MK, Ryu YL, Jung KH, Lee H, Lee HS, Yan HH (2013). HS-173, a novel PI3K inhibitor, attenuates the activation of hepatic stellate cells in liver fibrosis. Sci Rep.

[34] Talwalkar J (2010). Antifibrotic therapies—emerging biomarkers as treatment end points. Nat Rev Gastroenterol Hepatol.

[35] Son G, Hines IN, Lindquist J, Laura W, Schrum LW, Richard A, Rippe RA (2009). Inhibition of phosphatidylinositol 3-kinase signaling in hepatic stellate cells blocks the progression of hepatic fibrosis. Hepatology.

[36] Gracia-Sancho J, Marrone G, Fernández-Iglesias A (2019). Hepatic microcirculation and mechanisms of portal hypertension. Nat Rev Gastroenterol Hepatol.

[37] Ezhilarasan D (2020). Endothelin-1 in portal hypertension: the intricate role of hepatic stellate cells. Exp Biol Med.

[38] Xu W, Lu C, Zhang F, Shao J, Yao S, Zheng S (2017). Dihydroartemisinin counteracts fibrotic portal hypertension via farnesoid X receptor-dependent inhibition of hepatic stellate cell contraction. FEBS J.

[39] Edwards C, Feng HQ, Reynolds C, Mao L, Rockey DC (2008). Effect of the nitric oxide donor VPYRRO/NO on portal pressure and sinusoidal dynamics in normal and cirrhotic mice. Am J Physiol Gastrointest Liver Physiol.

[40] Gracia-Sancho J, Laviña B, Rodríguez-Vilarrupla A, García-Calderó H, Bosch J, García-Pagán JC (2007). Enhanced vasoconstrictor prostanoid production by sinusoidal endothelial cells increases portal perfusion pressure in cirrhotic rat livers. J Hepatol.

[41] Steib CJ, Gerbes AL, Bystron M (2007). Kupffer cell activation in normal and fibrotic livers increases portal pressure via thromboxane A(2). J Hepatol.

