# Supplementary material for: Kinin B1 receptor blockade attenuates hepatic fibrosis and portal hypertension in chronic liver diseases in mice
Source: J Transl Med. 2022 Dec 13;20:590. doi: 10.1186/s12967-022-03808-7 (PMC9746183; doi:10.1186/s12967-022-03808-7)
Supplement: Supplementary file 1 — Additional file 1. Figure S1. Study schema for (A) Carbon tetrachloride (CCl4) induced liver fibrosis and (B) Bile duct ligation (BDL) induced liver fibrosis. Figure S2. Densitometric analysis of western blots for protein expression of αSMA, Col1, VEGF, PCNA & P-AKT / AKT in (A) CCl4 and (B) BDL mice liver. Figure S3. Densitometric analysis of western blots for protein expression of COX-2, MCP-1, CD-68, and NE in (A) CCl4 and (B) BDL mice liver. Figure S4. FACS dot plots quantification graphs of Fig. 6C, F. Figure S5. (A) Cell Cycle flowcytometric plots quantification graphs of Fig. 8E; (B) Densitometric analysis of western blots (Fig. 8F) for protein expression of B1R, PCNA and phosphorylation of pP70s6 kinase, pPI3K, pGSK3 and pERK in LX-2 cells treated with DBK and BI113823. Figure S6. Schematic illustration of kinin B1 receptors in the pathogenesis of liver fibrosis. Table S1. Materials used in PCR. Table S2. Materials, primary and secondary antibodies. [file 12967_2022_3808_MOESM1_ESM.docx]

Supplementary data for Kinin B1 Receptor Blockade Attenuates Hepatic Fibrosis and Portal Hypertension in Chronic Liver Diseases in Mice

Dileep Reddy Rampa, Huiying Feng, Sivakumar Allur-Subramaniyan, Kwanseob Shim, Anton Pekcec, Dongwon Lee, Henri Doods, Dongmei Wu

|  | **Table of Contents** |  |
| --- | --- | --- |
| **1** | **Methods** | **1** |
| **2** | **Supplementary Figures** | **2** |
|  | **Supplementary Fig. 1.** | **2** |
|  | **Supplementary Fig. 2.** | **10** |
|  | **Supplementary Fig. 3.** | **11** |
|  | **Supplementary Fig. 4.** | **12** |
|  | **Supplementary Fig. 5.** | **13** |
|  | **Supplementary Fig. 6.** | **14** |
| **3** | **Supplementary Tables** | **15** |
|  | **Supplementary Table 1** | **15** |
|  | **Supplementary Table 2** | **16** |
| **4** | **References** | **17** |

#

# Methods

**BI113823, a novel potent and selective B1 receptor antagonist**

BI113823 is a novel potent and selective B1 receptor antagonist that exhibits high affinity for human, rat and mice B1 receptors (1, 2). BI-113823 inhibits the B1 receptor-cyclic adenosine monophosphate formation with a half maximal inhibitory concentration value of 19.1 nM, and it exerts analgesic properties in several animal models. It dose-dependently reversed the effects observed in Freund’s adjuvant (CFA) model, the weight bearing deficit in the monoiodoacetate model, and mechanical hyperalgesia in the carrageenan model (1). The compound has no affinity for the B2 receptor (IC50 >10.000 nM) and proved to be highly selective versus a large panel other receptors/enzymes or channels. Especially, we examined whether BI113823 directly interacts with angiotensin receptor or has an effects on blood pressure in rats. BI 113823 is devoid of an interaction with the angiotensin receptor and does not influence blood pressure in conscious rats in doses exceeding those used in present study.

**Carbon tetrachloride (CCl_4_) induced liver fibrosis**

Mice were randomly assigned to three study groups: 1) sham control, 2) CCl_4_ + vehicle, 3) CCl_4_ + BI 113823. Liver fibrosis was induced by intraperitoneal administration of CCl_4_ (1 ml/kg/body weight dissolved in olive oil [1:3]) twice a week for 6 weeks. Sham control mice received only olive oil injection. Mice received vehicle (0.1% Natrosol, p.o.) and BI 113823 (50mg/kg, p.o., a gift from Boehringer Ingelheim Pharma KG, Biberach, Germany) daily for 6 weeks. The dose was selected based on the pharmacokinetics and pharmacodynamics of this compound (information provided by Boehringer Ingelheim).

**Bile duct ligation (BDL) induced liver fibrosis**

Mice were anesthetized with ketamine (100 mg kg^-1^, i.m.) plus xylazine (10 mg kg^-1^, i.m.). A 2 cm abdominal midline incision was made and the common bile duct was located and tightly ligated with 4-0 silk suture. Sham control mice underwent identical laparotomy without BDL. Mice received daily treatment of vehicle (0.1% Natrosol, p.o.) and BI 113823 (50mg/kg, p.o.) for 3 weeks.

**Supplementary Figure S1**. Study schema for (A) Carbon tetrachloride (CCl_4_) induced liver fibrosis and (B) Bile duct ligation (BDL) induced liver fibrosis.

**Portal vein pressure measurements**

At the end of study protocol, mice were anesthetized as described above, an abdominal midline incision was made, and the portal vein was cannulated through an ileocolic vein with a 24-gauge catheter which was connected to a pressure transducer. Portal vein pressure was recorded using a Powerlab data acquisition system (ADInstruments Inc., CO). At the end of the experiment, liver tissues were collected. Tissue samples were snap frozen in liquid nitrogen or fixed in buffered formalin for histopathological examination.

**Histopathological Examination**

Liver tissue specimens were fixed in 10% formalin, processed with series of increasing ethanol (EtOH) concentrations and then embedded in paraffin, and sliced into 5-μm-thick sections. After deparaffinization, slides were stained with hematoxylin and eosin (HE) and picrosirius red and were examined for morphological alterations and collagen accumulation in liver by light microscopy. Fibrosis was quantified by Image J software. All histologic studies were performed in a blinded fashion.

**Immunohistochemical Staining:**

Immunohistochemical staining for α-SMA/B1R, CD68 and NE was performed to depict fibrosis and macrophage accumulation in liver tissue sections. Liver sections were deparaffinized, hydrated, and incubated in 10 mM sodium citrate buffer at 99°C for 20 minutes for antigen retrieval. Blocked sections were incubated with primary antibodies (Supplementary Table 2) overnight, followed by incubation for 1 hour with Fluorescence labeled secondary antibody (Supplementary Table 2). Sections were counterstained with Ultra Cruz Mounting Medium with DAPI (sc-24941; Santa Cruz Biotechnology) and covered by a coverslip. Fluorescent images were obtained by a Nikon Eclipse TE2000-U fluorescence microscope (Nikon, Tokyo, Japan) and a Nikon LWD 0.52 digital camera and intensities for α-SMA/B1R, CD68 and NE were quantified using Image Pro Premier 9.1 software.

**Hydroxy proline Assay:**

Hydroxyproline assay for total collagen content was performed according manufacturer instructions (biovision kit). Small fragments of different lobes were pooled, lyophilized and hydrolyzed in 12 N HCl at 120°C for 3hrs. Hepatic hydroxyproline content was spectrophotometrically measured using Ehrlich's reagent and the results expressed as ng/*μ*g of liver tissue protein.

**Western blotting**

Western blot experiments were performed as described previously (12). Briefly, total protein extracts were prepared by RIPA buffer and concentrations measured by BCA kit. 30ug of protein was separated by SDS PAGE and blotted onto PVDF membrane by semidry transfer unit (Biorad). Blots were incubated with primary Abs against B1R, B2R, COX-2, MCP-1, CD68, NE, α-SMA, Col1, VEGF, PCNA, AKT, p-AKT and GAPDH (Santa Cruz Biotechnology) followed by donkey anti-mouse HRP conjugated and goat anti-rabbit HRP conjugated secondary Abs. Immunoreactivity was detected using enhanced chemiluminescence autoradiography (ECL), and signals were scanned using iBrightCL1000 scanner and densitometrically quantified with ImageJ software.

**RNA isolation and quantitative real-time RT-PCR**

RNA was isolated from different groups of mouse lungs using an RNeasy Mini kit (QIAGEN, Valencia, CA), and cDNA was generated from 100 ng total RNA using MultiScribe reverse transcriptase (Applied Biosystems, Foster City, CA) and random and oligo-dT primers. Real-time quantitative PCR was performed in an AB 7500 sysytem using equal amounts of cDNA with TaqMan gene expression master mix or PowerUp SYBR Green Master mix (Applied Biosystems) and specific primers for the genes of interest (Supplementary Table 1). The average of each gene cycle threshold (Ct) was determined for each experiment. Relative cDNA levels (22DDCt) for the genes of interest were determined using the comparative Ct method, which generates DDCt as the difference between the gene of interest and the housekeeping genes 18s rRNA or GAPDH for each sample. Each averaged experimental gene-expression sample was compared with the averaged control sample, which was set to 1.

**Human peripheral** **blood immune cell assay**

*Immune cell migration assay*: Monocytes and neutrophils were isolated by Ficoll density gradient centrifugation from human peripheral blood samples from 12 healthy donors. These were then treated with TNF-α (5 ng/ml) in the presence or absence of BI 113823 (0.1, 1 µM). 1x10^6^ treated cells (in 0.5 ml serum free-RPMI medium) were added to upper chamber of transmigration plate (#140656 for monocytes, #140654 for neutrophils, Thermo Scientific, [Waltham,](https://en.wikipedia.org/wiki/Waltham,_Massachusetts) MA, USA) and 1.0 ml of serum free-RMPI media containing the same concentrations of TNF-α and the BI 113823 as in upper chambers were added to lower chambers. Cells were incubated at 37°C and 5% CO2for 12 hours, before collection from the lower chamber and counting by hemocytometer

*TNF-α production in human peripheral monocytes***:** human peripheral monocytes (2x10^6^) were seeded in 12 well plates with 2 ml serum free-RPMI media and treated with 10 ng/ml LPS in the presence or absence of BI 113823 (0.1 & 1 µM). Cells were incubated at 37°C and 5% CO2for 12 hours, then culture medium was collected and the levels of tumor necrosis factor (TNF)-α in the medium were measured using enzyme immunoassay kits for human TNF-α (PeproTech, Rocky Hill, NJ, USA).

*Myeloperoxidase (MPO) assay*: human peripheral neutrophils (2x10^6^) were seeded in 12 well plates with 1 ml serum free-RPMI media and treated with 5 ng/ml LPS in the presence or absence of BI 113823 (0.1 & 1 µM). Cells were incubated for 6 hours, the culture medium was collected, and MPO released by neutrophils was measured by determining the oxidation of o-dianisidine in the presence of hydrogen peroxide in a spectrophotometer at 460 nm.

*Human monocyte and neutrophil activation and respiratory burst***:** Activation of human monocytes and neutrophils was determined by flow cytometry by measuring cell surface molecule up-regulation of CD11b and CD18 on a FACSCalibur apparatus. Briefly, the cells were treated with LPS (25 ng/ml) in the presence or absence of B1R antagonist BI 113823 (0.1 µM), and incubated at 37°C and 5% CO2 for 12 hours. Cells were then incubated with FITC Mouse Anti-Human CD18 and APC Mouse Anti-Human CD11b/Mac-1 or the corresponding isotype controls (All from BD Pharmingen) for 20 mins in dark. After washing with PBS, the cells were analyzed for fluorescence intensity with a BD FACSCalibur apparatus. Data was analyzed using FlowJo_V10 software.

**LX2 hHSC assays**

*Lx-2 Cell culture*: LX2 hHSCs were kindly provided by Prof. Dr. Kim, Bumseok (Iksan Campus - Jeonbuk National University, South Korea). LX-2 cells were grown in DMEM medium supplemented with 10% FBS & antibiotics in T75 tissue culture flask at 37°C in a 5% CO2 humidified incubator.

*HSC Proliferation assay*: Cell proliferation in culture of LX2 hHSCs was determined by BrdU assay. HSCs were plated into 96-well microtiter plates (5000 cells/well) in growth medium. After 24 hours, medium was replaced with growth medium without or with different concentrations of des-Arg9-bradykinin (DBK) and BI 113823. After 3 days incubation, BrdU incorporation into DNA was determined using BrdU Cell Proliferation Assay Kit (Bio Vision Catalog # K306). Triplicate wells were used for each concentration and the experiment was repeated three times.

*HSC scratch wound assay*: HSCs were plated in 6-well culture plates until full confluence and starved overnight in 0.5% FBS containing medium. A standardized scratch was made using a 200 μl pipette tip. Cells were washed and incubated with 2 ml of 0.5% FBS containing medium with different concentrations of DBK and BI 113823 to measure the migratory response of the cells into the scrape areas, microscopic photographs were taken at 0 h and 24 h. Images were analyzed by NIH ImageJ software to calculate the area of scratch wound healed.

HSC *contraction assay*: The effects of BI 113823 on TGF-β mediated HSC contraction was evaluated using cytoselect 48-well cell contraction assay kit (cell biolabs, Catalog #CBA-5021). Photographs were made with a digital camera at 0, 24, and 48 hrs. The size of the gels were digitally measured and normalized with their respective well size using Image J software.

*HSC Apoptosis assay*: HSCs were treated with BI 113823 at different concentrations for 48 h in medium containing 1% FBS, and cells were collected and labeled with Annexin V-FITC and propidium iodide (PI) for flow cytometry analysis according to manufacture instructions (FITC Annexin V Apoptosis Detection Kit with PI, Biolegend).

*Cell cycle analysis*: HSCs were plated in 6-well culture plates and starved overnight in serum free medium. Medium was replaced with medium containing 1% FBS, without or with DBK and BI 113823 for 24 hours. Cells were fixed with ice-cold 70% ethanol, labeled with PI, and followed by flow cytometry analysis as described in Fxcycle PI/RNase staining kit manual (thermofisher # F10797)

*HSC* *Migration Assay*: HSC transwell migration assay was performed using an 8 μm pore size transmigration plate (Thermo Scientific – 140656). The lower chambers were filled with 1ml serum free medium in control, medium with 1% FBS in vehicle, and medium with 1% FBS+ BI 113823 in drug group. HSC s (1 × 10^6^ cells) were added in the upper chamber in 0.5ml serum free medium and incubated in CO2 incubator. After 12 hours non-migrating cells were removed from the upper surface by a cotton swab. The cells that migrated through the membrane to the lower surface were removed by trypsinization and then polled and counted under a microscope using hemocytometer.

*Immunohistochemical Staining for α‐SMA*: HSCs were plated in glass bottom confocal dishes, treated for 24 hrs with TGFβ and BI 113823, and were fixed with methanol. Blocked dishes were incubated with α‐SMA primary Ab overnight. Followed by three washes with PBST and incubation for 1 hour with rhodamine fluoresce probe–labeled secondary antibody and washed thrice. Glass bottoms were removed slowly and mounted on slide with hard-set mounting medium with DAPI. Fluorescence images were obtained by a Zeiss LSM 880 Confocal microscope.

*Western blot*: Western blot was performed as above using secondary antibodies as mentioned in Supplementary Table 2. The bands were visualized using ECL detection reagent and photographed using iBrightCL1000 scanner. Intensity of individual bands was quantified using ImageJ densitometry software, and expressed in% relative to GAPDH.

# Supplementary Figures

## Supplementary Fig. S2.

Densitometric analysis of western blots for protein expression of αSMA, Col1, VEGF, PCNA & P-AKT / AKT in (A) CCl4 and (B) BDL mice liver. Data are representative of three independent experiments, with n = 7-8. *p* values indicated in panels, *ns*. not significant and significant as *p < 0.05; **p < 0.01; ***p < 0.001. One-way ANOVA and Tukey’s multiple comparison test were performed.

## Supplementary Fig. S3.

Densitometric analysis of western blots for protein expression of COX-2, MCP-1, CD-68, and NE in (A) CCl4 and (B) BDL mice liver. Data are representative of three independent experiments, with n = 7-8. *p* values indicated in panels, *ns*. not significant and significant as *p < 0.05; **p < 0.01; ***p < 0.001. One-way ANOVA and Tukey’s multiple comparison test were performed.

## Supplementary Fig. S4.

**A**

**B**

(FACS dot plots quantification graphs of Figure 6C, 6F). BI113823 treatment reduced LPS induced CD11/CD18 expression in (A) human monocytes and (B) human neutrophils. Data are representative of three independent experiments, with n = 3. *p* values indicated in panels, *ns*. not significant and significant as *p < 0.05; **p < 0.01; ***p < 0.001. Two-way ANOVA and Bonferroni post tests were performed.

**Supplementary Fig. S5.**


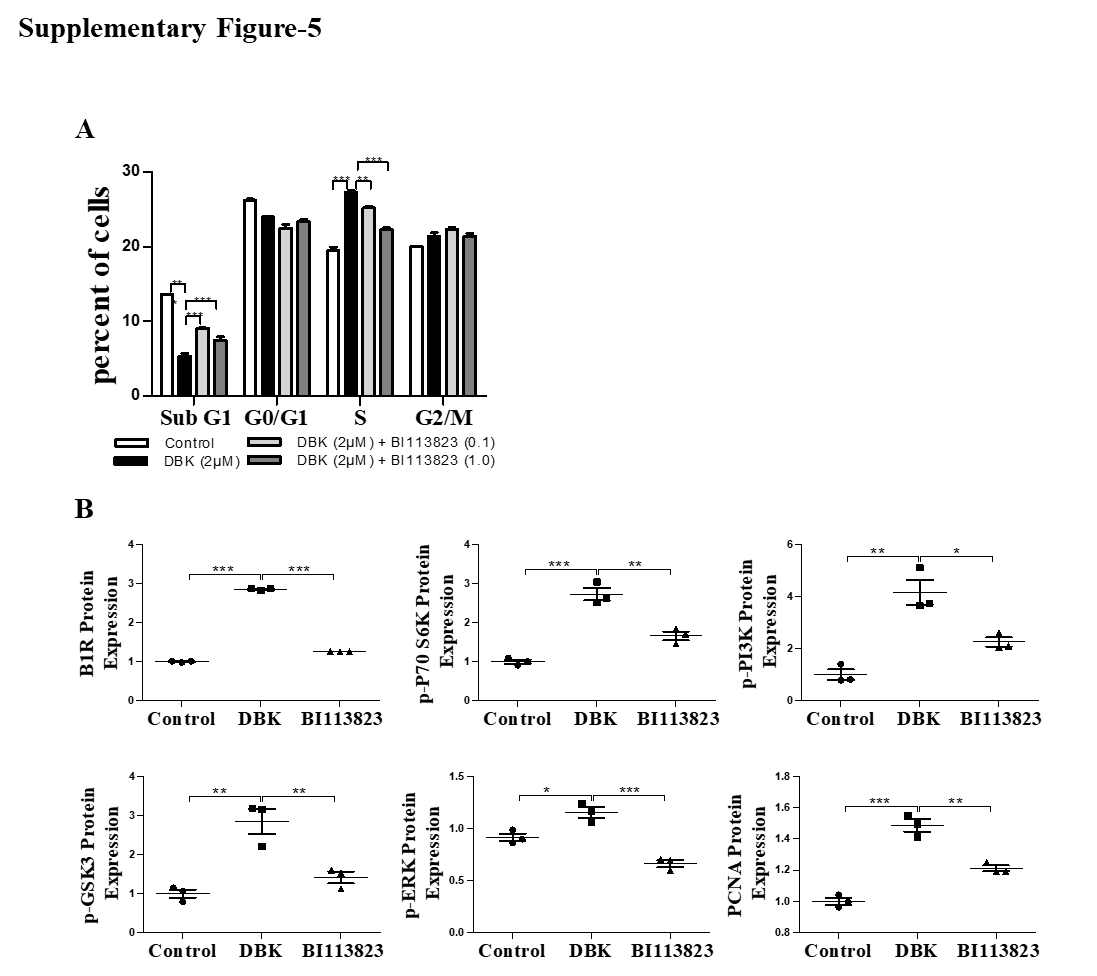


1. Cell Cycle flowcytometric plots quantification graphs of figure 8E. BI113823 treatment reduced DBK induced cell cycle progression from G0 to G1 and G1 to S phase.
2. Densitometric analysis of western blots (Figure 8F) for protein expression of B1R, PCNA and phosphorylation of pP70s6 kinase, pPI3K, pGSK3 and pERK in LX-2 cells treated with DBK and BI113823. Data are representative of three independent experiments, with n = 3. p values indicated in panels, ns. not significant and significant as *p < 0.05; **p < 0.01; ***p < 0.001. Two-way ANOVA and Bonferroni post tests were performed.

**Supplementary Fig. S6.**

**Schematic illustration of kinin B1 receptors in the pathogenesis of liver fibrosis.**


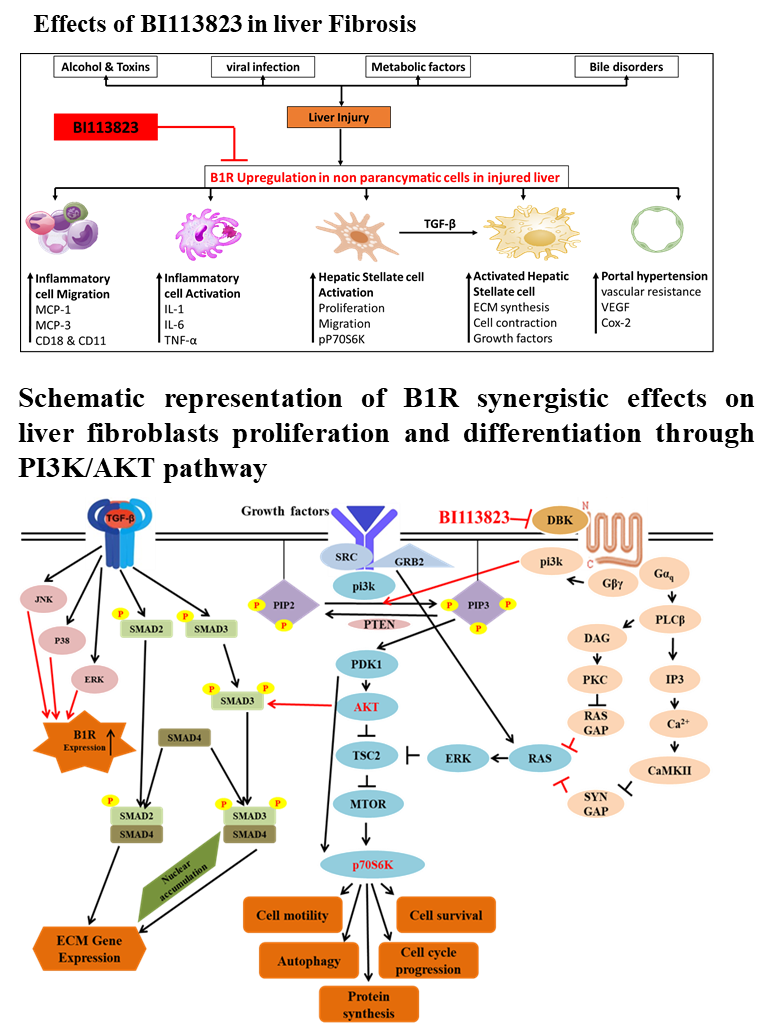


# Supplementary Tables

## Supplementary Table 1

## Supplementary Table 2

**References**

1. Doods H, Hauel N, Kirsten A, Kramer G, Ceci A.BI 113823, a novel B1

receptor antagonist exhibiting antinociceptive properties in inflammatory pain models. Pain Practice. 2012; 12:18.

1. [Wu](https://www.ncbi.nlm.nih.gov/pubmed/?term=Wu%20D%5BAuthor%5D&cauthor=true&cauthor_uid=23236443) D, [Lin](https://www.ncbi.nlm.nih.gov/pubmed/?term=Lin%20X%5BAuthor%5D&cauthor=true&cauthor_uid=23236443) X, [Bernloehr](https://www.ncbi.nlm.nih.gov/pubmed/?term=Bernloehr%20C%5BAuthor%5D&cauthor=true&cauthor_uid=23236443) C, [Hildebrandt](https://www.ncbi.nlm.nih.gov/pubmed/?term=Hildebrandt%20T%5BAuthor%5D&cauthor=true&cauthor_uid=23236443) T, and [Doods](https://www.ncbi.nlm.nih.gov/pubmed/?term=Doods%20H%5BAuthor%5D&cauthor=true&cauthor_uid=23236443) H. Effects of a Novel Bradykinin B1 Receptor Antagonist and Angiotensin II Receptor Blockade on Experimental Myocardial Infarction in Rats. [PLoS One.](https://www.ncbi.nlm.nih.gov/pmc/articles/PMC3517424/) 2012; 7(12): e51151.
